# Developing Microbial Co-Culture System for Enhanced Polyhydroxyalkanoates (PHA) Production Using Acid Pretreated Lignocellulosic Biomass

**DOI:** 10.3390/polym14040726

**Published:** 2022-02-14

**Authors:** Rijuta Ganesh Saratale, Si-Kyung Cho, Avinash Ashok Kadam, Gajanan Sampatrao Ghodake, Manu Kumar, Ram Naresh Bharagava, Sunita Varjani, Supriya Nair, Dong-Su Kim, Han-Seung Shin, Ganesh Dattatraya Saratale

**Affiliations:** 1 Research Institute of Biotechnology and Medical Converged Science, Dongguk University-Seoul, Ilsandong-gu, Goyang-si 10326, Gyeonggi-do, Korea; rijutaganesh@gmail.com (R.G.S.); avikadam2010@gmail.com (A.A.K.); 2 Department of Biological and Environmental Science, Dongguk University, Ilsandong-gu, Goyang-si 10326, Gyonggi-do, Korea; sk.cho@dongguk.edu (S.-K.C.); ghodakegs@gmail.com (G.S.G.); 3 Department of Life Science, Dongguk University-Seoul, 32 Dongguk-ro, Ilsandong-gu, Goyang-si 10326, Gyeonggi-do, Korea; manukumar007@gmail.com; 4 Department of Environmental Microbiology, School for Environmental Sciences Babasaheb Bhimrao Ambedkar University (A Central University), Lucknow 226 025, Uttar Pradesh, India; bharagavarnbbau11@gmail.com; 5 Gujarat Pollution Control Board, Gandhinagar 382 010, Gujarat, India; drsvs18@gmail.com; 6 Department of Research and Development, SRL Limited, Prime Square, S. V. Road, Goregaon (W), Mumbai 400 062, Maharashtra State, India; suprianair@gmail.com; 7 Department of Environmental Science and Engineering, Ewha Womans University, Seoul 120-750, Korea; dongsu@ewha.ac.kr; 8 Department of Food Science and Biotechnology, Dongguk University-Seoul, Ilsandong-gu, Goyang-si 10326, Gyeonggi-do, Korea; spartan@dongguk.edu

**Keywords:** *Lysinibacillus* sp. RGS, *Ralstonia eutropha* ATCC 17699, co-culture strategy, sugarcane bagasse, acid pretreatment, polyhydroxyalkanoates production

## Abstract

In the growing polymer industry, the interest of researchers is captivated by bioplastics production with biodegradable and biocompatible properties. This study examines the polyhydroxyalkanoates (PHA) production performance of individual *Lysinibacillus* sp. RGS and *Ralstonia eutropha* ATCC 17699 and their co-culture by utilizing sugarcane bagasse (SCB) hydrolysates. Initially, acidic (H_2_SO_4_) and acidified sodium chlorite pretreatment was employed for the hydrolysis of SCB. The effects of chemical pretreatment on the SCB biomass assembly and its chemical constituents were studied by employing numerous analytical methods. Acidic pretreatment under optimal conditions showed effective delignification (60%) of the SCB biomass, leading to a maximum hydrolysis yield of 74.9 ± 1.65% and a saccharification yield of 569.0 ± 5.65 mg/g of SCB after enzymatic hydrolysis. The resulting SCB enzymatic hydrolysates were harnessed for PHA synthesis using individual microbial culture and their defined co-culture. Co-culture strategy was found to be effective in sugar assimilation, bacterial growth, and PHA production kinetic parameters relative to the individual strains. Furthermore, the effects of increasing acid pretreated SCB hydrolysates (20, 30, and 40 g/L) on cell density and PHA synthesis were studied. The effects of different cost-effective nutrient supplements and volatile fatty acids (VFAs) with acid pretreated SCB hydrolysates on cell growth and PHA production were studied. By employing optimal conditions and supplementation of corn steep liquor (CSL) and spent coffee waste extracted oil (SCGO), the co-culture produced maximum cell growth (DCW: 11.68 and 11.0 g/L), PHA accumulation (76% and 76%), and PHA titer (8.87 and 8.36 g/L), respectively. The findings collectively suggest that the development of a microbial co-culture strategy is a promising route for the efficient production of high-value bioplastics using different agricultural waste biomass.

## 1. Introduction

**Figure 1:**
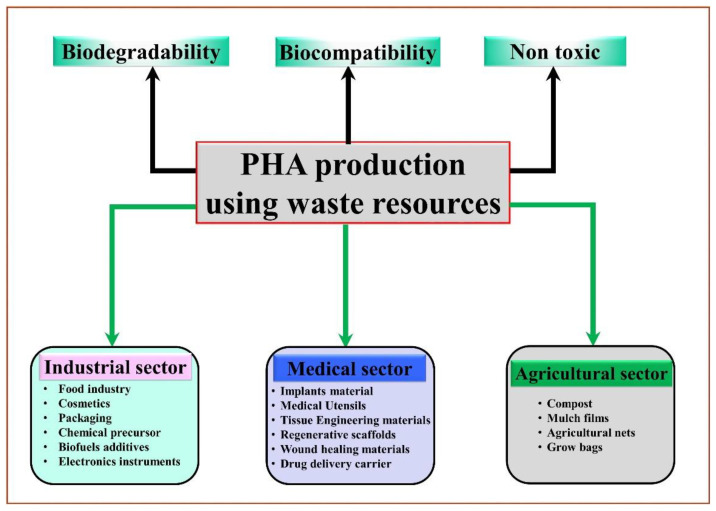
Potential applications of PHA in different industrial sectors.

Synthetic plastics have multiple applications in the modern world such as in food packaging, automotive industry, sports materials, agricultural, biomedical apparatus, and electronic materials. Global plastic production extended to 359 million metric tons in 2018, equated to 200 in 2002 and 50 in 1976 [1]. However, being resistant to degradation, large amounts of plastic waste discarded primarily in open landfills and dumpsites inflict severe impacts on the natural ecosystem, human health, and the environment [2,3]. Furthermore, conventional thermal recycling of used plastic products also has limitation of releasing toxic residues, such as dioxins, hydrogen chloride, and sulfur oxides, during their degradation [4]. These critical issues have sparked an immediate response from the global community for producing alternatives with functionally similar material that are readily biodegradable without compromising workability and convenience [5,6]. Polyhydroxyalkanoates (PHA) are microbial polyesters and have great potential for developing sustainable and eco-friendly materials such as bioplastics [7]. PHA is biosynthesized by the polymerization of hydroxyalkanoates (HAs), wherein the hydroxyl (–OH) groups are usually present at the β-carbon of the polymer [8]. A wide range of bacteria can accumulate PHAs as granules in the cytoplasm with a size of approximately 0.2–0.5 μm under an inadequate quantity of nutrients and the availability of rich carbon sources [9]. Their excellent physicochemical properties, such as toughness, strength, flexibility, and thermo-mechanical characteristics, make PHA a promising and sustainable alternative to synthetic plastics [7,9,10]. PHA is widely utilized in various industries, such as pharmaceuticals, health, and agriculture, for many applications (Figure 1).

Although microbes capable of producing PHA are naturally present in various environments, only a few are efficient enough to produce these polyesters with high production rates and efficiency [11]. Some of the most common and industrially essential microbes studied for PHA production include *Ralstonia eutropha*, *Bacillus megaterium*, *Lysinibcaillus* sp., *Cupriavidus nector*, *Pseudomonas aeruginosa*, and *Pseudomonas fluorescens* [5,6,10]. There are many techno-economic challenges for commercial-scale PHA production, primarily high production cost and limited productivity. The carbon source is one of the foremost issues distressing PHA production at an industrial scale, since it directly affects cell growth, production efficiency, molecular mass, superiority, and configuration [11,12]. To overcome these issues, various waste feedstock materials, for example, waste fluxes generated from food, milk, and sugar processing industries and agricultural residues, were attempted for PHA synthesis [5,11]. In addition to this, to enhance PHA production, various strategies, including isolation of new robust production strains, improvement of the strains employing genetic engineering, feedstock selection, fermentation technology, and bioreactor design, have been well studied [6,12].

Moreover, mixed bacterial cultures are extensively considered for PHA production using different waste biomass resources. This process, which does not require sterile conditions, can utilize waste substrates effectively and further converts into PHA, making the process cost-effective [13]. However, in this PHA production process, sustaining microbial concentrations and governing the optimum constancy of the microorganisms in the fermentation media are complex [14]. During the extraction of juice from sugarcane, a fibrous residue of sugarcane bagasse (SCB) equivalent to 540 million metric tons per year globally is generated [15]. To make the process more economical, eco-friendly, and sustainable in this study, abundant SCB has been selected as a potential carbon substrate for PHA production. However, direct consumption of SCB by microorganisms is problematic due to the fact of their compact structure and the presence of lignin. Therefore, various physical, chemical, and physicochemical pretreatment methods have been studied in hydrolysis of lignocellulosic biomass [16]. Recently, some investigators showed the potential of innovative green solvents, mainly ionic liquids and deep eutectic solvents for the pretreatment of lignocellulosic biomass. The results are satisfactory but some challenges still remain including their cost, recyclability, effects of water, and limits their applications at the large scale [17,18]. Acid pretreatment was found to be efficient for hydrolysis and delignification of biomass, but it produces various inhibitors during this process which adversely affects the enzymatic hydrolysis and microbial fermentation processes. Because of this, the effective assimilation of biomass and PHA production using pure microbial culture are difficult. Considering this, we developed a defined co-culture of *Ralstonia eutropha* ATCC 17699 and isolated *Lysinibacillus* sp. RGS, which are well-recognized PHA producers [10,19]. This study assessed the capability of defined co-culture using chemically pretreated SCB hydrolysates as a probable carbon substrate for PHA production. Co-culture studies also assessed the effects of increasing SCB hydrolysates and supplementation of cost-effective nutrients and VFAs to enrich cell growth and PHA synthesis. The development of a co-culture strategy could be helpful for the effective utilization of SCB and significant PHA production by which the process becomes sustainable, cost-effective, and eco-friendly.

## 2. Materials and Methods

### 2.1. Biomass and Chemicals

Sugarcane bagasse was collected from the local sugar industry, GS-Caltex, South Korea. The substrate was dried to eradicate moisture and a constant weight was attained. The dried biomass was further sheared into small pieces, sieved to obtain a particle size of approximately 0.5 mm, and stored at room temperature. The other required chemicals used in the experimentations were of high purity analytical grade (AR).

### 2.2. Chemical Pretreatment of SCB and Enzymatic Hydrolysis

Acid pretreatment was conducted by combining SCB with H_2_SO_4_ with 1% (*w/v*) concentration at 121 °C for 15 min, whereas acidified sodium chlorite pretreatment was performed adopting 0.4 g sodium chlorite and 0.2 mL acetic acid per gram of SCB with periodic mixing at 80 °C in a fume hood [20]. In each pretreatment, the ratio of SCB biomass to the liquid phase was retained at 1:10. The resulting pretreated SCB biomass was washed thoroughly with water as long as the pH of the reaction solution was neutral. Each pretreated biomass was separated using vacuum filters and dried at 60 °C till a persistent weight was attained. The untreated (autoclaved at 121 °C for 15 min without any chemical agent) and chemically pretreated SCB biomass was assessed immediately for its chemical composition by employing previously described standard methods [21]. The structural configuration changes of untreated and each chemically treated SCB biomass were observed. Images were taken by SEM JEOL JSM-6360A microscope (JEOL, Tokyo, Japan) using the standard protocol. Fourier transform infrared (FTIR) spectra of SCB biomass were performed using FTIR spectroscopy (Cary 630; Agilent, Santa Clara, CA, USA). The spectra were documented from 4000 to 400 cm^−^^1^ through the typical scan of 16 scans at a resolution of 4 cm^−^^1^. X-ray diffraction (XRD) examination of SCB biomass was performed with a scanning rate (2°/min) in the range of 10°–50° by using D2 Phaser tabletop model at 30 kV (Bruker, Billerica, MA, USA). The crystallinity changes during chemical pretreatment were determined by quantifying the crystallinity index (CrI) using the standard protocol [22].

### 2.3. Enzymatic Hydrolysis of Pretreated SCB

Enzymatic saccharification of untreated and each chemically pretreated SCB biomass was conducted in 100 mL Erlenmeyer conical flask containing 2.0% (*w/v*) biomass in 20 mL of 50 mM citrate buffer (pH 5.0) with 0.005% (*w/v*) sodium azide and the enzyme loading of cellulase from *Trichoderma reesei* ATCC 26921 of 20 FPU/g of SCB. The reaction solution was further placed at 50 °C for 24 h under shaking conditions (150 rpm). The sample aliquots were withdrawn at the function of time and measured saccharification yield in terms of reducing sugar production after enzymatic hydrolysis by standard dinitrosalicylic acid technique [23]. In addition, the overall hydrolysis and glucose yields were assessed by following the standard methodology reported earlier [10]. The resulting SCB enzymatic hydrolysates were concentrated by heating the solution at 80 °C, followed by centrifugation and PHA production.

### 2.4. Development of Microbial Co-Culture of Lysinibacillus sp. and Ralstonia eutropha

The strain *Ralstonia eutropha* ATCC 17699 was obtained from ATCC (Manassas, VA, USA) whereas *Lysinibacillus* sp. RGS was isolated from the soil [19]. Pure culture of *R. eutropha* and *Lysinibacillus* sp. was preserved on nutrient agar slants. Later, *R. eutropha* and *Lysinibacillus* sp. were grown in a 250 mL Erlenmeyer flask containing 100 mL tryptic soy bbroth without dextrose (TSB; Becton Dickinson) and nutrient broth ((g/L): beef extract, 3; peptone, 10; NaCl), respectively. Both cultures were cultivated at 37 °C for 36 h, under shaking environments (100 rpm). Afterwards, cells were collected by centrifugation (5400× *g*; Hanil, Seoul, Korea) followed by washing two times with phosphate-buffered saline (PBS) solution. The microbial co-culture of *R. eutropha* and *Lysinibacillus* sp. was developed by aseptically transferring the 1.0 mL suspension of each preculture transferred to the PHA fermentation media. While for individual culture, 2.0 mL suspension was transferred to the PHA production medium to maintain the same cell count in the monoculture and defined co-culture. The PHA production medium had the following composition (g/L) with addition of SCB biomass hydrolysate with designated quantities underneath: NaH_2_PO_4_, 3.6; Na_2_HPO_4_, 2.84; K_2_SO_4_, 3.486; NaOH, 0.4; yeast extract, 0.2; MgSO_4_·7H_2_O, 0.39; CaCl_2_, 0.062; (NH_4_)_2_SO_4_, 0.1; CuSO_4_·5H_2_O, 0.005; ZnSO_4_·7H_2_O, 0.024; MnSO_4_·H_2_O, 0.024; FeSO_4_·7H_2_O, 0.15; pH 7.0.

### 2.5. PHA Production Using Chemically Pretreated SCB Hydrolysates by Individual and Co-Culture Microbial System

Initially, the isolated *Lysinibacillus* sp. and *R. eutropha* and their defined co-culture were inoculated in a PHA production medium containing each chemically pretreated SCB hydrolysates with a concentration of 20 g/L. The inoculated flasks were incubated at 37 °C under shaking conditions (100 rpm) for 48 h. In addition, the PHA production performance of microbial co-culture was investigated by taking an elevated concentration of 1% H_2_SO_4_ pretreated SCB hydrolysates (20, 30, and 40 g/L). Lastly, the effects of nutrient and volatile fatty acid supplementation with 1% H_2_SO_4_ pretreated SCB hydrolysates (20 g/L) to accelerate bacterial growth and PHA accumulation using microbial co-culture were thoroughly examined. The bacterial cell progress and PHA production kinetics parameters were studied using the earlier detailed procedure. The dry cell weight of pure and co-culture during fermentation was performed by collecting the cells by centrifugation. The obtained cell pellets were washed with hexane and distilled water. The resulting cell pellets were later lyophilized, and then the dry cell weight (DCW) was measured. After 48 h of fermentation, the bacterial cells were separated. PHA was extracted from lyophilized bacterial cell powder by dispersion of chloroform in a sodium hypochlorite solution followed by its retrieval using 80% methanol precipitation and filtration method [19].

The cell growth and PHA production parameters were calculated by employing the following formulas:

(1) Residual biomass (g/L)=Dry cell weight (DCW) - Extracted quantity of PHA (g/L)

(2) $$\mathrm{PHA}\;\mathrm{accumulation}\;(\%)=\frac{\mathrm{Extracted}\;\mathrm{quantity}\;\mathrm{of}\;\mathrm{PHA}\;(g/L)}{\mathrm{Dry}\;\mathrm{cell}\;\mathrm{weight}\;(g/L)\;}\times \;100$$

(3) $${\mathrm{PHA}\;\mathrm{productivity}\;(Q}_{p})=\frac{\mathrm{PHA}\;\mathrm{final}\;\mathrm{quantity}\;(g/L)}{\mathrm{Fermentation}\;\mathrm{period}\;(48\;h)\;}$$

(4) $${\mathrm{PHA}\;\mathrm{yield}\;\mathrm{coefficient}\;\mathrm{owing}\;\mathrm{to}\;\mathrm{cell}\;\mathrm{biomass}\;(Y}_{p/b})=\frac{\mathrm{PHA}\;\mathrm{final}\;\mathrm{quantity}\;(g/L)}{\mathrm{Dry}\;\mathrm{cell}\;\mathrm{weight}\;(g/L)}$$

(5) $${\mathrm{PHA}\;\mathrm{yield}\;\mathrm{coefficient}\;\mathrm{owing}\;\mathrm{to}\;\mathrm{substrate}\;\mathrm{consumption}\;(Y}_{p/s})=\frac{\mathrm{PHA}\;\mathrm{final}\;\mathrm{quantity}\;(g/L)}{\mathrm{SCB}\;\mathrm{hydrolysates}\;\mathrm{utilized}\;(g/L)}$$

### 2.6. Statistical Analysis

The obtained results were determined using one-way analysis of variance (ANOVA) followed by Tukey’s HSD test in the GraphPad InStat version 3.06 software GraphPad Software Inc., San Diego, CA, USA). A threshold of *p* = 0.05 was deliberated significantly to evaluate differences between means.

## 3. Results and Discussion

### 3.1. Preparation of Sugarcane Bagasse Feedstock for PHA Production

Lignocellulosic biomass (LC) was reflected as an abundant, sustainable, and cost-effective carbon substrate for PHA production. It was estimated that accessible LC biomass is approximately 150 billion tons per year globally [24]. However, the presence of lignin and the structural complexity of LC biomass are limiting factors for its efficient utilization. Thus, for the effective exploitation of LC biomass and its enzymatic saccharification, it is obligatory to develop chemical pretreatment methods to disrupt the structure of LC biomass. Moreover, PHA production economies using LC biomass relies on a substrate, choice of pretreatment, hydrolysis method, and fermentation conditions. Acid pretreatment was found to be an effective chemical pretreatment method that leads to solubilization of hemicellulose content and removal of lignin from the biomass. Additionally, acid chlorite pretreatment, a mixture of sodium chlorite and acetic acid (ASC), was found to be competent for removing lignin with a lower loss of polysaccharide components of biomass [25].

**Figure 2:**
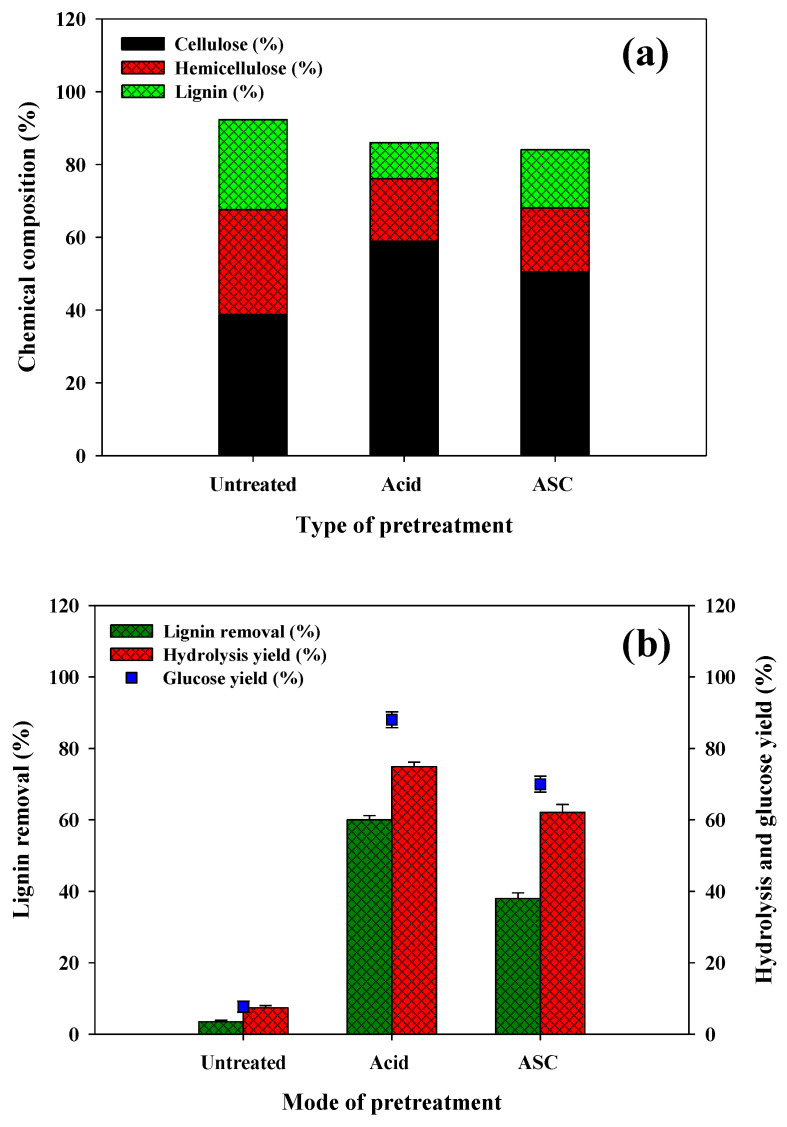
Effects of acidic (1% H_2_SO_4_) and acidified sodium chlorite pretreatment on the (**a**) chemical composition and (**b**) saccharification yield of sugarcane bagasse.

Sugarcane bagasse is one of the abundant byproducts of sugar industries, having 30–50% cellulose content and, thus, can be deliberated as a suitable substrate for PHA production [26]. Initially, SCB was exposed to acid (1% H_2_SO_4_) and ASC pretreatment, wherein acid pretreatment was found effective in lignin removal (60%) compared to ASC pretreatment (36.5%). There was a sharp upsurge in cellulose content from 38.8% to 58.9% and 50.5% in acid and ASC pretreatment, respectively (Figure 2a).

The results confirmed that acid (1% H_2_SO_4_) pretreatment was effective for the delignification of SCB by which cellulose and hemicellulose components became more accessible for enzymatic hydrolysis. The details of the biochemical constituents of SCB before and after each chemical pretreatment are presented in Figure 2a and Table 1. Enzymatic hydrolysis of pretreated biomass and effective saccharification are vital factors for lucrative PHA production. Acid (1% H_2_SO_4_) pretreated biomass gave a significant saccharification yield (569.0 ± 5.65 mg/g of SCB) with substantial hydrolysis yield (74.9 ± 1.65%) and glucose yield (87.8 ± 1.14%) (Figure 2b, Table 1). The attained saccharification yield of SCB (74.9 ± 1.65%) appeared to be greater relative to sulphuric acid pretreated SCB (30.7%) [27].

### 3.2. Analytical Characterization of Chemically Pretreated SCB Biomass

**Figure 3:**
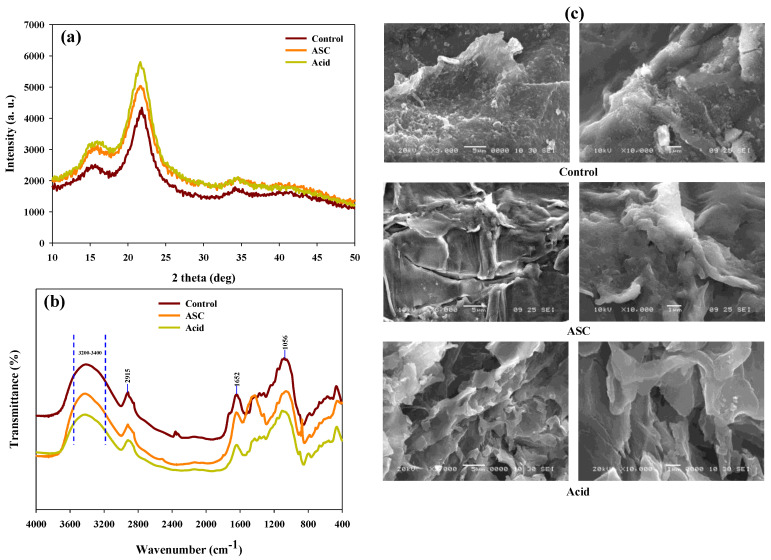
(**a**) X-ray diffraction pattern; (**b**) FTIR spectra; (**c**) SEM micrographs of sugarcane bagasse before and after ASC and acid (1% H_2_SO_4_) chemical pretreatment.

The modifications in crystallinity, chemical functional groups, and exterior structure of SCB after each chemical pretreatment were studied using standard XRD, FTIR, and SEM analytical tools. XRD is a vital analytical tool to determine changes in the crystalline index (CrI) suited to dictate the efficiency of selected pretreatment for the hydrolysis of SCB. After acid pretreatment, a sharp escalation in CrI was observed; conversely, a modest improvement in CrI was recorded in ASC pretreatment (Figure 3a). The results suggest that acid pretreatment is significant for lignin removal and exposes the cellulose content for the enzymatic hydrolysis. Similar observations were observed in lime pretreated SCB and acid pretreated corn stover [28,29]. FTIR spectrum of untreated and each pretreated SCB was recorded. An increase in width and symmetry in the region of 3200 and 3400 cm^−^^1^ indicates the dissociation of the cellulosic structure of SCB biomass (Figure 3b), whereas changes in the peak intensity at 2915 cm^−^^1^ indicate –CH_2_ stretching and rupture of cellulose [30].

Similarly, a sharp decrease in intensity at approximately 1652 cm^−^^1^ demonstrated lignin removal, since this peak was attributed to the stretching vibration of aromatic rings and phenyl ester side-chain C=O bonds of lignin (Figure 3b). The other peak at 1056 cm^−^^1^ changed, referring to the removal of amorphous cellulose [16]. The surface morphological changes were studied using SEM. In the SEM images of untreated SCB, the surface was smooth and precise, whereas in acid pretreated SCB, the surface was rougher and became more permeable than ASC pretreatment (Figure 3c). The surface developed porous attributable to higher dissociation of SCB biomass. The analytical results suggest that in acid pretreatment, there was significant elimination of lignin and hemicellulose by which cellulose content is highly available for enzymatic hydrolysis resulting in superior saccharification yield.

### 3.3. PHA Production Studies

The global bioplastics market demonstrates incredible growth in manufacturing sustainable products for various applications. The production of PHA using various lignocellulosic biomass is especially advantageous in achieving simultaneous resource recovery and pollution prevention, and it makes the process fruitful and eco-friendly. However, during acid and ASC pretreatment of SCB, some toxic compounds, for instance, furfural and hydroxymethylfurfural, formic, acetic acid, and various lignin degraded aromatics, are generated, which directly influences the microbial growth during fermentation and consequently PHA production [31,32]. To overcome the effects of fermentation inhibitors in the SCB hydrolysates, many investigators have proposed some solutions, mainly, adaptation of potential microbial strains to the inhibitors [31,33]; detoxification of hydrolysates before PHA production [34]; addition of a higher quantity of inoculum size for effective PHA production [27]. *Ralstonia eutropha* showed less capability to utilize 5C sugars and oligosaccharides in LC biomass hydrolysates. However, this strain exhibited the potential to grow on the fermentation inhibitors generated during pretreatment and to produce PHA [27]. Furthermore, some *Bacillus* species showed the ability for higher assimilation of xylose relative to glucose for their growth and PHA production [35,36]. A co-culture system is widely utilized for bioremediation of contaminants, wastewater treatment, bioenergy, and value-added chemicals production [37,38]. For the efficient utilization of chemically pretreated LC biomass hydrolysates and PHA production, using individual culture has limitations. It was observed that the buildout of microbial co-culture of different species with diverse metabolic activities would be a viable solution for effective LC biomass to PHA conversion accompanied by desired co-polymers production [39,40,41].

**Figure 4:**
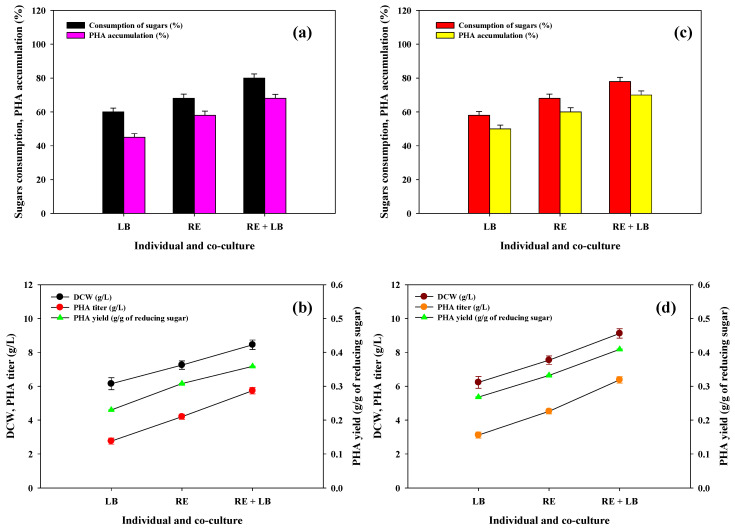
Assimilation of sugar, growth, and PHA productions kinetics parameters by using monoculture (LB: *Lysinibacillus* sp. RGS; RE: *Ralstonia eutropha*) and their co-culture using (**a**,**b**) ASC and (**c**,**d**) acid (1% H_2_SO_4_) pretreated SCB enzymatic hydrolysates (each 20 g/L concentration).

Considering this perspective, this study demonstrated the viability of producing PHA polymers using monoculture of *R. eutropha*, *Lysinibacillus* sp., and their defined co-culture using chemically pretreated SCB enzymatic hydrolysates. During this study, SCB enzymatic hydrolysates of each chemical pretreatment were utilized without applying a detoxification procedure for PHA production. By keeping the acid and ASC pretreated SCB hydrolysate concentration constant (20 g/L), individual *Ralstonia eutropha* and *Lysinibacillus* sp. showed less sugar consumption and cell growth and PHA accumulation compared to the co-culture. Figure 4 portrays the cell growth and PHA production kinetics parameters using monoculture and co-culture using ASC and acid pretreated SCB hydrolysates. Maximum sugar consumption was observed in ASC pretreated (80%) relative to acid pretreated (78%) SCB enzymatic hydrolysates, although PHA accumulation is higher in acid pretreated hydrolysates (Figure 4a,c). The maximum DCW (8.45 and 9.12 g/L), PHA accumulation (68 and 70 %), and PHA titer (8.45 and 9.12 g/L) were observed in ASC and acid pretreated hydrolysates by applying a co-culture system (Figure 4b,d). The synergistic metabolic activities between microbial co-culture counteract internal and external distresses, leading to superior substrate utilization and PHA production [42,43]. The PHA synthesis and bacterial cell growth were found to be greater in acid pretreated SCB hydrolysates, so further investigation was carried out using the acid pretreated SCB hydrolysates. The initial results confirm that the developed co-culture systems were found to be efficacious for higher assimilation of SCB hydrolysates and PHA production; nonetheless, more research is still required to understand the exact mechanism.

#### 3.3.1. Effects of Substrate Concentration

Substrate concentration is an important parameter that directly influences microbial metabolic activities, microorganisms’ substrate consumption, and their growth coupled with PHA production. Thus, optimization of substrate concentration is essential to attain maximum cell growth and PHA synthesis. For that reason, different concentrations of acid pretreated SCB hydrolysates (20, 30, and 40 g/L) were systematically investigated on co-culture growth and PHA synthesis. The results have been presented in Table 2. The outcomes proposed that co-culture can efficiently assimilate sugars with higher growth and PHA production up to 30 g/L of acid pretreated hydrolysates. However, with a further increase in hydrolysate concentration, no considerable growth and PHA production was recorded. These inhibitory effects are conceivably due to the osmotic pressure from the higher concentration of sugar and fermentation inhibitors in SCB hydrolysates. Similar observations were observed in other PHA production studies specifically; using sugarcane molasses by *Bacillus megaterium* strain [44]; using horticultural waste hemicellulosic hydrolysate by isolated *Candida athensensis* SB18 [45] and using corn stover by *Paracoccus* sp. LL1 [46].

#### 3.3.2. Effects of Cost-Effective Nutrients Supplementation in PHA Production Media

**Figure 5:**
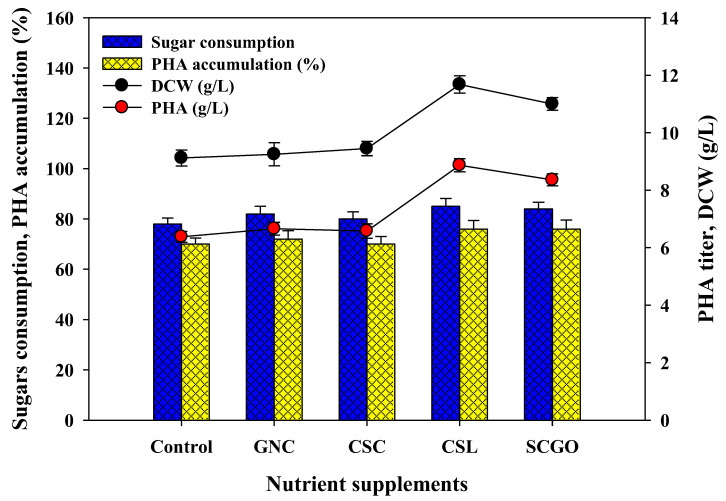
Effects of supplementation of inexpensive nutrient supplements with acid pretreated SCB enzymatic hydrolysates (20 g/L) on sugar assimilation, growth, and PHA productions kinetics parameters by defined microbial co-culture of *Lysinibacillus* sp. RGS and *Ralstonia eutropha*.

To make the LC biomass to PHA production process economical and sustainable, higher assimilation of LC hydrolysates with greater cell densities and volumetric productivities is needed. Considering this viewpoint, acid pretreated hydrolysates (20 g/L) with cost-effective nutrient supplements (1%) such as cottonseed cake (CSC) and groundnut cake (GNC), corn steep liquor (CSL), and spent coffee ground extracted oil (SCGO) were evaluated to improve cell density and PHA production. Among the nutrient supplements, CSL and SCGO were found to be beneficial in increasing cell growth (28% and 20%), PHA synthesis (8.5% and 8.5%), and PHA yield (39% and 31%) relative to control without any nutrient supplementation (Figure 5). At the same time, other nutrient supplements including GNC and CSC were found not to be productive for both parameters. Worldwide millions of tons of spent coffee grounds (SCG) waste product are generated from coffee consumption [47]. It was supposed that the bioactive compounds of the CSL and SCGO could be advantageous for microbial growth and PHA production. Obruca et al. [48] explored the potential of *Cupriavidus necator* H16 for the successful production of PHA using spent coffee oil as a substrate. The initial results recommend that supplementation of CSL and SCGO is favorable to attain maximum cell density and for large-scale LC biomass PHA production.

#### 3.3.3. Effects of Individual Volatile Fatty Acid Supplementation in PHA Production Media

**Figure 6:**
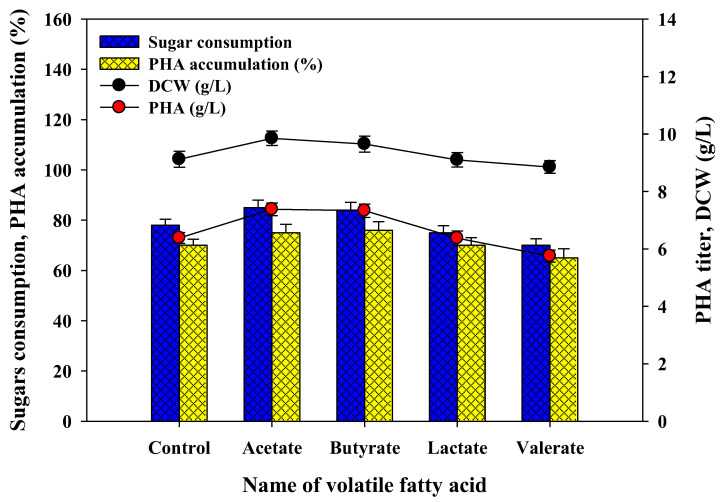
Effects of supplementation of individual volatile fatty acid with acid pretreated SCB enzymatic hydrolysates (20 g/L) on sugar assimilation, growth, and PHA productions kinetics parameters by defined microbial co-culture of *Lysinibacillus* sp. RGS and *Ralstonia eutropha*.

After anaerobic digestion of waste biomass, biogas, and acidogenic effluents (VFAs; mainly acetate, butyrate, propionate, and valerate) are generated, leading to environmental pollution. The utilization of VFA as a supplement in PHA production makes the process more successful and alleviates environmental problems. In the case of supplementation of individual VFA in the fermentation media, acetate and butyrate can induce bacterial growth (8.0% and 5.8%) and PHA production (15.6% and 14.8%) as compared to the control without any VFA (Figure 6). It was supposed that acetate and butyrate may act as intermediate metabolites which induce the PHA production pathway, leading to an increase in bacterial cell growth and PHA synthesis. However, lactate was found to be less productive, and valerate showed inhibitory results in both parameters (Figure 6). To understand the exact mechanism of VFA in cell growth and PHA production, more research is still required. In other studies, the supplementation of organic and inorganic nitrogen sources, nutrient supplements, and VFAs were found to be productive in PHA synthesis [47,49,50,51].

Moreover, the obtained DCW, PHA accumulation, and PHA titer by defined co-culture were higher than other PHA production studies utilizing sugarcane bagasse biomass as a potential substrate. The details are provided in Table 3.

## 4. Conclusions

In this work, an effective acid pretreatment was used for the preparation of SCB feedstock and further utilized for PHA production. The strategic approach for the development of co-culture stimulated biomass growth with a synchronized increase in the polymer accumulation using SCB hydrolysates relative to individual microbial culture. Moreover, supplementation of CSL and SCGO was found productive to achieve higher cell density with significant PHA productivities. In conclusion, the results suggest that applying co-culture of potential PHA producing strains with diverse metabolic activities could be considered as a viable option to achieve higher PHA productivities. This approach can open new possibilities in developing the performance of microbial PHA in sustainable biorefinery concepts utilizing different lignocellulosic biomass and its further development. Additionally, research should be directed towards understanding the molecular mechanism of enhanced PHA production, cost-effective detoxification procedure and application of the developed process at an industrial level by designing a suitable bioreactor.

## Figures and Tables

**Table 1:** Effect of acidic (1% H_2_SO_4_) and acidified sodium chlorite (ASC) pretreatment on chemical components and saccharification of sugarcane bagasse.

Pretreatment	Biochemical Components (%)			On Enzymatic Hydrolysis	
	Cellulose	Hemicellulose	Lignin	Enzymatic Hydrolysis Yield (%)	Total Reducing Sugar
No pretreatment	38.80 ± 0.85	28.82 ± 0.68	24.70 ± 0.68	7.44 ± 0.32	50.0 ± 1.38
Acid (1% H_2_SO_4_)	58.91 ± 1.28	17.22 ± 0.42	9.82 ± 0.32	74.90 ± 1.65	569.0 ± 5.65
ASC	50.50 ± 0.88	17.54 ± 0.41	16.02 ± 0.44	62.10 ± 1.58	422.2 ± 4.98

Values are the mean of three experiments ± SEM. One-way ANOVA determined statistics with Tukey–Kramer multiple comparisons test.

**Table 2:** Assimilation of sugar, growth, and PHA productions kinetics parameters utilizing different concentrations of acid pretreated SCB enzymatic hydrolysates (20, 30, and 40 g/L) by co-culture of *Lysinibacillus* sp. RGS and *Ralstonia eutropha*.

Parameters	Acid Pretreated SCB Enzymatic Hydrolysates Concentration (g/L)		
	20	30	40
Fermentation period (h)	48	48	48
Total Sugar assimilation (%)	78.0 ± 1.54	84.0 ± 1.65	80.0 ± 1.72
Dry cell weight (DCW, g/L)	9.12 ± 0.38	14.24 ± 0.65	16.32 ± 0.71
Residual biomass (g/L)	2.74 ± 0.28	3.99 ± 0.32	5.23 ± 0.26
PHA accumulation (%)	70.0 ± 2.50	72.1 ± 2.15	68.2 ± 1.98
PHA titer (g/L)	6.38 ± 0.25	10.25 ± 0.42	11.09 ± 0.52
Q_p_ g PHA/L/h	0.132 ± 0.001	0.213 ± 0.001	0.231 ± 0.001
PHA yield (g/g)	0.409 ± 0.001	0.406 ± 0.001	0.346 ± 0.001

Values are the mean of three experiments; (±) standard error; (SE) by one-way ANOVA with Tukey–Kramer multiple comparisons test.

**Table 3:** Comparison of cell growth and PHA accumulation by different microbial strains using sugarcane bagasse as a potential substrate.

Name of Substrate	Type of Pretreatment	Microorganism	Operation Mode	PHA Accumulation (%)	PHA Concentration (g/L)	Reference
Sugarcane bagasse	Ultrasound + alkaline pretreatment	*Lysinibacillus* sp. RGS	Batch	61.5	5.31	[19]
Sugarcane bagasse	Acid pretreatment	*Ralstonia eutropha*	Batch 50:50	56.7	6.06	[27]
Sugarcane bagasse	Acid pretreatment	*Burkholderia glumae* MA13	Batch	14.95	0.09	[31]
Sugarcane bagasse	Acid pretreatment	*Burkholderia* sp. F24	Fed-Batch	49.31	12.25	[33]
Sugarcane bagasse	Acid pretreatment Detoxified	*Burkholderia cepacia* IPT 048	Fed-batch	53	2.3	[34]
Sugarcane bagasse	Acid pretreatment + Detoxified	*Burkholderia sacchari* IPT 101	Fed batch	62	2.7	[34]
Sugarcane bagasse	Acid pretreatment	*Halogeometricum borinquense* strain E3	Batch	50.4	1.6	[47]
Sugarcane bagasse	Biological pre-treatment with *Pycnoporus coccineus* MScMS1	*Bacillus megaterium* Ti3	Batch	65	0.58	[52]
Sugarcane bagasse	Acid pretreatment	*Bacillus thuringiensis*	Batch	39.6	4.2	[53]
Sugarcane bagasse	Acid pretreatment	*R. eutropha +Lysinibacillus* sp. Co-culture	Batch	70.0	6.38	This study
